# Trauma outcomes in nonfatal road traffic accidents: a Portuguese medico-legal approach

**DOI:** 10.1080/20961790.2022.2031548

**Published:** 2022-03-23

**Authors:** Flávia Cunha-Diniz, Tiago Taveira-Gomes, José Manuel Teixeira, Teresa Magalhães

**Affiliations:** a CINTESIS—Faculty of Medicine, University of Porto, Porto, Portugal; b IINFACTS—Institute of Research and Advanced Training in Health Sciences and Technologies, Department of Sciences, University Institute of Health Sciences (IUCS), CESPU, CRL, Gandra, Portugal; c Fernando Pessoa University, Porto, Portugal; d Porto Healthcare Unity—Accidents, Fidelidade—Insurance Company, Porto, Portugal

**Keywords:** Forensic sciences, traffic accident, trauma, injury, damage, outcome assessment, legal medicine

## Abstract

The objective of this study was to compare the outcomes of nonfatal road traffic accidents by the victims’ age group and sex. We used the Portuguese medico-legal rules for personal injury assessment, in the scope of the Civil Law in that country, which includes a three-dimensional methodology. This was a retrospective study including 667 victims of road traffic accidents aged 3–94 years old. Their final medico-legal reports all used the Portuguese methodology for personal injury assessment. Outcomes were analysed by the victims’ age group (children, working-age adults, and older people) and sex. Road traffic accidents were generally serious (ISS mean 9.5), with higher severity in children and older people. The most frequent body sequelae were musculoskeletal (64.8%), which were associated with functional and situational outcomes. Temporary damage resulted in an average length of impairment of daily life of 199.6 days, 171.7 days to return to work, and an average degree of quantum doloris (noneconomic damage related to physical and psychological harm) of 3.7/7. The average permanent damage was 7.3/100 points for Permanent Functional Deficit, 0.43/3 for Permanent Professional Repercussion, 2/7 for Permanent Aesthetic Damage, 3.9/7 for Permanent Repercussion on Sexual Activity and 3.2/7 for Permanent Repercussion on Sport and Leisure Activities. Overall, 19% of people became permanently dependent (10.6% needed third-party assistance). The medico-legal methodology used, considering victims’ real-life situation, allows a comprehensive assessment. There were several significant differences among the three age groups but none between sexes. These differences and the impact of the more severe cases justify further detailed medico-legal studies in these specific situations on children, older people, and severely injured victims.

**Key points:**
- This was a retrospective study of accident mechanisms and injury outcomes in Portugal, and considered the outcomes in the victims’ real-life situation.
- Lesions from road traffic accidents were generally serious, with higher severity among children and older people.
- The most frequent sequels were musculoskeletal, and associated with functional and situational outcomes.
- Both temporary and permanent outcomes had repercussions for the victims.
- There were significant differences between children, working-age adults and older people, but none between sexes.

## Introduction

Road traffic accidents (RTAs) are a global health, social and economic problem that cause up to 50 million injuries each year [1]. In Portugal, in 2019, there were 35 704 accidents involving victims, and 474 fatal and 45 503 nonfatal injuries [2]. The non-fatal injuries affected 442.4 victims/100 000 inhabitants. In 2020, these accidents decreased significantly because of the pandemic.

RTA survivors experience short- and long-term health consequences, sometimes leading to impairment and disability, with considerable economic costs that may have a major impact on their quality of life and their families [3]. Personal outcomes depend on the characteristics of the accident (e.g. pedestrians, cyclists and motorcyclists are more likely to be severely injured [3, 4]), the characteristics of the victim (e.g. age, sex and previous health condition), and the type and severity of lesions, which seem to be the primary predictive factor for the outcome of the trauma [4]. However, only a few medico-legal studies examine RTA outcomes analysed from a comprehensive and personalised perspective, and consider temporary, permanent, economic and noneconomic outcomes.

Personal injury assessment (PIA) in legal medicine may offer a detailed and personalised description and quantification of trauma outcomes. However, medico-legal methodologies, including the damage parameters that are assessed, differ with national legislative systems [5]. In many countries, no official guidelines are available for this assessment. In Portugal, there are rules for PIA dictated by the National Institute of Legal Medicine and Forensic Sciences. These rules are set out in Civil Law and followed by both public and private services. They include the following [6, 7]:

1. A *three-dimensional (3D) methodology* for a comprehensive description of any permanent damage [8, 9]. This method offers a systematic and validated solution to describe and analyse, in an eco-systemic way, the consequences of a specific trauma on physical and psychological integrity and health. It considers three personal levels: (a) the *body* level assesses biological outcomes that may include morphological, anatomical, histological, physiological and even genetic particularities; (b) *capacities* (or *functions*) assess physical and mental capacities (current or potential), taking into account the age and sex, irrespective of the life setting; (c) *life situations* (or *participation and activities*) assess the confrontation (concrete or potential) between those affected and the reality of their physical, familial, social, cultural, educational and professional environment.
2. The damage parameters (Table 1), which consider temporary and permanent outcomes, including economic and noneconomic aspects, assessed using the Portuguese rules [6, 7, 9].

**Table 1.** Portuguese medico-legal damage parameters.

Damage parameters	Meaning and evaluation criteria
Temporary professional repercussion	Economic temporary damage: period (days) in which the victim is unable to perform his/her usual professional activity.
Total temporary functional deficit	Noneconomic temporary damage: period (days) in which the victim is prevented from autonomously performing acts of daily, family, and social life (without any reference to professional activity). Mostly corresponds with hospitalisation time.
Partial temporary functional deficit	Noneconomic temporary damage: period (days) in which the victim may resume activities of daily, family, and social life with some degree of autonomy, although still with limitations.
Quantum doloris	Noneconomic temporary damage: physical and psychic suffering experienced by the victim during the period of temporary damage on a 7-points scale of increasing severity.
Permanent professional repercussion	Economic permanent damage: victim’s ability to perform professional activity. Levels: 0—Without work affected; 1—Additional effort for usual work or need for workplace adaptation or use of technical aids; 2—Total incapacity for work in the scope of his/her technical-professional qualifications, with need of professional reconversion; 3—Total incapacity for any kind of work.
Permanent functional deficit	Noneconomic permanent damage: definitive effects on the victim’s physical and/or psychic integrity, with repercussion on daily life activities, including family and social life, leisure, and sporting activity, although it is independent of professional activities. Assessed by the *National Permanent Disability Table* (Annex 2 of the Decree-Law no. 352/2007, 23rd October); 100-points scale of increasing severity.
Future damage	Damage that is not yet observable in the PIA, but whose development is sure, corresponding to an aggravation of the sequelae, in the future, and consequent aggravation of certain damage parameters, namely, *Permanent Functional Deficit*.
Permanent aesthetic damage	Noneconomic permanent damage: repercussion of the sequelae upon the victim’s self-image and image from others on a 7-points scale of increasing severity.
Permanent repercussion on sexual activity	Noneconomic permanent damage: total or partial limitation on the level of sexual performance/gratification arising from the physical and/or psychic sequelae on a 7-points scale of increasing severity.
Permanent repercussion on sporting and leisure activities	Noneconomic permanent damage: impossibility of the victim engaging in certain leisure, physical or social activities which he/she did regularly, and which represented a clear source of personal fulfilment and gratification on a 7-points scale of increasing severity.
Permanent dependences	Economic permanent damage: it corresponds to the victim’s needs, with repercussion on his/her independence and autonomy; it should be assessed considering the victim’s best chances of rehabilitation and reintegration.

PIA, personal injury assessment.

The objective of this study was therefore to compare the outcomes of nonfatal RTAs, considering the victims’ age group and sex, using the Portuguese medico-legal rules for PIA.

### Materials and methods

This was a retrospective study using a convenience sample based on medico-legal reports of PIA cases. The reports’ inclusion criteria were as follows: (a) final medico-legal report about victims of RTAs (we did not consider the victim’s age and sex, the accident type, severity of injuries, or type of insurance responsibility—with or without fault—at this stage), showing that the causality nexus between the trauma and damage was established; (b) performed at a healthcare unit of a Portuguese insurance company because this includes the majority of these reports; (c) occurring between 2018 and 2019; and (d) performed by the same physician, to assure data reliability and considerable experience of the Portuguese official rules, and the use of the 3D methodology for describing permanent outcomes [6, 7, 9] and the different levels and types of damage, for quantifying outcomes (Table 1) [6, 7]. This gave a total of 667 cases, and three age groups were considered: (a) children (<18 years) (*n =* 56; 8%); (b) working-age adults (18–64 years) (*n =* 431; 65%); and (c) older people (>64 years) (*n =* 180; 27%).

The *Injury Severity Score* (ISS) was used for retrospective estimation of injury severity [10, 11] in the acute phase, using the clinical files. The ISS variables were categorised in classes as 0 (non-existent), 1–8 (minor or moderate), 9–15 (serious), 16–24 (severe), and 25–75 (critical). The *Inventory for Handicap Assessment* (IHA) was used to quantify the severity of damage at the body, functional and situational levels, and the damage coefficient [8], at the date of the PIA. This coefficient corresponds to the average of the final scores that results from each scale of the body, functional and situational levels and considers five severity groups (Table 2). *Permanent Functional Deficit* (PFD) was categorised as 0, 1–9, 10–19, 20–39 and 40–100, drawing on the case distribution and the severity groups.

**Table 2.** Meaning of the severity degree of body, functional and situational levels, and damage coefficient, considering the 3D methodology.

Degree	Body sequels	Functional and situational permanent outcomes	Damage coefficient
0	Non-existent	Without difficulties	Independence
1	Minimal	Minimum difficulties	Independence but slowness or discomfort
2	Mild	Medium difficulties	Dependence of either medicines or technical aid
3	Important	Important difficulties	Dependence of partial third-party assistance
4	Severe	Impossible	Dependence of total third-party assistance

A database was created for the study and completed by the physician who performed the medico-legal assessment of the cases. No information was included that could allow those involved to be identified. All analyses used SPSS for Windows Version 25.0 (IBM Corp., Armonk, NY, USA). Descriptive statistics were used to describe the study population, in total and stratified by age and sex. The chi-square test was used to assess the dependence between the frequency variables. Continuous variables were assumed to be normal, and tests for differences between variables were performed using Student’s *t*-test. In all analyses, the level of statistical significance was set at a *P*-value of <0.05.

## Results

The average timespan between RTA and the final PIA was 337.4 ± 421.9 days (min = 32; max = 4 476).

### Victim demographics

Overall, there were similar numbers of female victims (*n =* 334; 50.1%) and male victims (*n =* 333; 49.9%), but there were more women (103/180; 57.2%) among the older people (*P =* 0.05). The mean age at the date of RTA was 48.7 ± 21.5 years (min = 3; max = 94), with working-age adults being the main population (65%). Male victims were younger than female ones (*P =* 0.01), with mean ages of 46.6 ± 20.9 vs. 50.8 ± 22.0. Most people were professionally active at the date of the accident (*n =* 342; 51.3%), with the remainder being students (*n =* 63; 9.4%), stay-at-home spouses (*n =* 16; 2.4%), retired (*n =* 194; 29.1%), unemployed (*n =* 47; 7.0%), and preschool children (*n =* 5; 0.8%). The majority presented a pathological (*n =* 431; 64.6%) and/or traumatic history (*n =* 213; 31.9%), with significant differences between older people and adult victims for pathological cases (*P =* 0.05): 19.4% (35/180) vs. 10.0% (43/431).

### Accident characterisation

The majority of RTAs consisted of a motor vehicle impact (423/667; 63.4%), with the next-largest groups being pedestrians who were run over (*n =* 214) and bicyclists hit (*n =* 30) by a motor vehicle (244/667; 36.6%). In motor vehicle impact cases, cars were the most common vehicle (277/423; 65.5%), followed by motorcycle (109/423; 25.8%), then truck, tractor, or bus (37/423; 8.7%). Most of the motor vehicle impacts were crashes between vehicles (337/423; 79.7%), with the remaining being sideslips (52/423; 12.3%), victims falling inside a bus (22/423; 5.2%), and victims falling from the vehicle (4/423; 0.9%), with eight (1.9%) classified as “others”. The victim was the driver in 63.1% (267/423) of cases. Most victims wore protective devices at the time of the accident (363/415; 87.5%), including seat belts (254/306; 83.0%) or helmets (109/109; 100%). In cars with airbags (198/305; 64.9%), 49.0% (97/198) deployed.

Both children and older people were more likely to be run over than working-age adults (*P =* 0.03 and *P <* 0.001). However, working-age adults experienced more collisions in motor vehicles than children or older people (*P =* 0.01 and *P <* 0.001).

### Injury characterisation

Limbs were the most commonly injured body region (53.5% lower and 49.5% upper limbs) (Table 3). Only two victims experienced no physical injuries but complained of psychiatric distress due to the severity of the injuries suffered by other victims involved in the RTA, including one death. There were differences between the age groups in injury distribution.

**Table 3.** Injury location and severitya.

Items		Total (*N =* 667) *n* (%)	Children (*n =* 56) *n* (%)	Adults (*n =* 431) *n* (%)	Older people (*n =* 180) *n* (%)	Adults *vs*. children (*P*)	Adults *vs*. older people (*P*)
Injury location^b^	Lower limbs	357 (53.5)	30 (53.6)	232 (53.8)	95 (52.8)	0.97	0.81
	Upper limbs	330 (49.5)	21 (37.5)	219 (50.8)	90 (50.0)	0.006*	0.86
	Head and neck	258 (38.7)	20 (35.7)	155 (36.0)	83 (46.1)	0.97	0.02*
	Chest and abdomen	172 (25.8)	9 (16.1)	102 (23.7)	61 (33.9)	0.16	0.01*
	Spine/spinal cord	162 (24.3)	3 (5.4)	108 (25.1)	51 (28.3)	<0.001*	0.4
	Face	118 (17.7)	14 (25.0)	71 (16.5)	33 (18.3)	0.16	0.58
	Non-existent	2 (0.3)	1 (1.8)	1 (0.2)	0 (0)	–	–
Injury severity score	1–8 (minor/moderate)	362 (54.3)	26 (46.4)	267 (61.9)	69 (38.3)	0.03*	<0.001*
	9–15 (serious)	161 (24.1)	18 (32.1)	88 (20.4)	55 (30.6)	0.08	0.01*
	16–24 (severe)	72 (10.8)	6 (10.7)	38 (8.8)	28 (15.6)	0.64	0.03*
	≥25 (critical)	70 (10.5)	5 (8.9)	37 (8.6)	28 (15.6)	0.93	0.02*
	Non-existent	2 (0.3)	1 (1.8)	1 (0.2)	0 (0)	–	–

aPercentages may not total 100 due to rounding. bInjury location categories are not mutually exclusive.*P values indicating significant differences.

The mean ISS was 9.5 ± 9.8 (min *=* 0; max = 50), with 21.3% being severe or critical (≥16). The ISS was 12.0 ± 10.3 in older people, 9.6 ± 5.0 in children, and 8.5 ± 9.2 in working-age adults, with a significant difference between older people and working-age adults (*P =* 0.004). The ISS was significantly different between those who were run over and in collisions (11.8 ± 10.5 vs. 8.2 ± 9.3, *P <* 0.001). No differences were found by sex.

### Temporary outcomes

The medico-legal evaluation of temporary damage parameters is described in Table 4, with differences between age groups but not between sexes. *Quantum doloris* (QD) was attributed in all cases, and its degree of distribution was: (a) 1 (*n =* 1; 0.1%); (b) 2 (*n =* 36; 5.4%); (c) 3 (*n =* 244; 36.6%); (d) 4 (*n =* 278; 41.7%); (e) 5 (*n =* 86; 12.9%); (f) 6 (*n =* 21; 3.1%); and (g) 7 (*n =* 1; 0.1%). Differences were found between all temporary damage and ISS severity and between the type of accident and QD (Table 5).

**Table 4.** Parameters of damage among the three age groups (*N =* 667).

Damage parameters	Total		Children (mean ± SD)	Adults (mean ± SD)	Older people (mean ± SD)	Adults *vs*. children (*P*)	Adults *vs*. older people (*P*)
	Mean ± SD	Min–max					
Total temporary functional deficit (days)	23.8 ± 81.7	0–1 095	33.3 ± 148.7	18.1 ± 69.2	34.4 ± 79.5	0.03*	0.001*
Partial temporary functional deficit (days)	179.0 ± 212.2	0–2 101	215.4 ± 397.1	181.5 ± 196.3	161.7 ± 160.2	0.004*	0.005*
Temporary professional repercussion (days)	171.7 ± 208.9	0–1 252	–	–	–	–	–
Quantum doloris (1–7)	3.7 ± 0.9	1.0–7.0	4.0 ± 1.0	3.6 ± 0.0	4.0 ± 0.8	0.003*	<0.001*
Permanent functional deficit (0–100)	7.3 ± 12.3	0–100	4.9 ± 15.9	6.2 ± 11.0	10.9 ± 13.2	0.44	<0.001*
Permanent professional repercussion (0–3)	0.43 ± 0.7	0–3	–	0.4 ± 0.7	0.8 ± 1.0	–	0.08
Permanent aesthetic damage (1–7)	2.0 ± 1.0	1–6	2.3 ± 1.5	2.0 ± 1.0	1.8 ± 0.8	0.04*	0.73
Permanent repercussion on sexual activity (1–7)	3.9 ± 1.7	1–7	–	3.6 ± 1.5	4.5 ± 1.6	–	0.05*
Permanent repercussion on sport/leisure activities (1–7)	3.2 ± 1.8	1–7	4.0 ± 1.8	2.9 ± 1.7	3.6 ± 1.9	0.97	0.29
Damage coefficient (0–4)	2.0 ± 0.9	0–4	1.4 ± 0.7	1.8 ± 0.7	2.5 ± 1.1	<0.001*	<0.001*

* *P* values indicating significant differences.

**Table 5.** Correlations between injury severity score (ISS) severity and RTA type for different parameters of damage (*N =* 667).

	ISS			*P*	Type of accident		*P*
Damage parameters	<16				Collisions	Run overs	
Total temporary functional deficit	7.4 ± 33.0	84.5 ± 150.9		*<*0.001*	20.3 ± 87.9	31.2 ± 74.8	0.12
Partial temporary functional deficit	149.7 ± 174.9	287.1 ± 289.6		*<*0.001*	175.2 ± 208.9	203.3 ± 238.2	0.13
Temporary professional repercussion	139.5 ± 185.8	352.3 ± 239.2		*<*0.001*	169.9 ± 210.5	195.9 ± 228.2	0.35
Quantum doloris	3.5 ± 0.8	4.6 ± 0.8		*<*0.001*	3.6 ± 0.9	3.9 ± 0.8	<0.001*
Permanent functional deficit	3.8 ± 5.0	20.3 ± 20.1		<0.001*	6.7 ± 12.5	9.0 ± 12.6	0.03*
Permanent professional repercussion	1.1 ± 0.4	2.0 ± 1.2		<0.001*	1.4 ± 0.9	1.5 ± 1.0	0.6
Permanent aesthetic damage	1.7 ± 0.9	2.4 ± 1.2		<0.001*	2.0 ± 1.0	2.0 ± 1.1	0.5
Permanent repercussion sexual activity	2.0 ± 1.7	4.2 ± 1.5		0.03*	4.2 ± 1.4	3.6 ± 1.6	0.3
Permanent repercussion on sport/leisure activities	2.4 ± 1.7	3.6 ± 1.7		0.007*	2.9 ± 1.7	3.5 ± 1.8	0.2
Damage coefficient	1.7 ± 0.7	2.7 ± 1.1		<0.001*	1.8 ± 0.8	2.1 ± 1.0	<0.001*

### Permanent outcomes

The 3D evaluation of permanent damage is described in Table 6. The most frequent body sequelae were musculoskeletal (64.8%). They were associated with functional outcomes, primarily for carriage/displacement/transfers (52.0%), and with situational outcomes, particularly related to acts of daily life (51.7%). The majority of injuries had a 3D severity degree of 0–1 (non-existent or minimal) for (a) body sequelae (65.9%); (b) functional outcomes (88.0%); (c) situational outcomes (87.8%); and (d) damage coefficient (85.3%) (Table 7). Differences were observed between children and working-age adults (*P* = 0.001; *P* = 0.4; *P* < 0.001; and *P* < 0.001 for each degree) and between older people and working-age adults (all *P <* 0.001) but not between sexes. Differences were also observed between ISS and all types of severity degrees (all *P <* 0.001).

**Table 6.** Permanent outcome description from the three-dimensional evaluation (*N* = 667).

Permanent outcome description	*n* (%)
Body sequelae^a^	
Orthopaedic	432 (64.8)
Aesthetic	67 (10.0)
Neurologic	55 (8.2)
Psychiatric	33 (4.9)
Dermatologic	22 (3.3)
Otorhinolaryngologic	15 (2.2)
Angio-cardiologic	9 (1.3)
Stomatologic	9 (1.3)
Ophthalmologic	7 (1.0)
Gastroenterologic	6 (0.9)
Urologic	5 (0.8)
Others	3 (0.5)
Non-existent	169 (25.3)
Functional permanent outcomes^a^	
Carriage, displacement, and transfers	347 (52.0)
Manipulation and grip	197 (29.5)
Cognition and affectivity	126 (18.9)
Sphincter’s control	25 (3.7)
Communication	20 (3.0)
Sexuality	20 (3.0)
Senses	17 (2.5)
Others	71 (10.6)
Non-existent	183 (27.4)
Situational permanent outcomes^a^	
Acts of daily living	345 (51.7)
Affective and social life, sporting and leisure activities	270 (40.5)
Professional life or academic training	243 (36.4)
Non-existent	187 (28.0)

^a^Three-dimensional outcomes are not mutually exclusive.

**Table 7.** Severity degree of permanent outcomes, assessed using the three-dimensional methodology (*N =* 667, see Table 2).

Permanent outcomes	Severity degree, *n* (%)				
	0	1	2	3	4
Body	169 (25.3)	271 (40.6)	163 (24.4)	49 (7.4)	15 (2.3)
Functions	458 (68.7)	129 (19.3)	72 (10.8)	6 (0.9)	2 (0.3)
Situations	441 (66.1)	145 (21.7)	57 (8.5)	16 (2.4)	8 (1.2)
Damage coefficient	206 (30.9)	363 (54.4)	27 (4.0)	64 (9.6)	7 (1.0)

The evaluation of medico-legal permanent damage parameters is described in Tables 4 and 8. PFD (Table 8) was considered in 72.9% of cases (486/667), ranging from 1 to 19 points in 64.0% of cases (427/667), with differences between older people and working-age adults (*P <* 0.001) but not between sexes (Table 4). Correlations were also found between PFD and all types of severity degrees (all *P <* 0.001) and ISS (*P <* 0.001). It was considered that in cases with prior pathological history (*n =* 431), the previous state of the victim had influenced the accident outcomes, increasing the PFD value from an average of 5.8 ± 12.7 points to 8.2 ± 12 points (*P =* 0.02).

**Table 8.** Other permanent outcomes (*N* = 667).

Permanent outcomes	*n* (%)^b^
Permanent functional deficit (0–100 points)	
0	181 (27.1)
1–9	335 (50.2)
10–19	92 (13.8)
20–39	38 (5.7)
40–100	21 (3.1)
Permanent professional repercussion	
0	242 (36.3)
1	103 (15.4)
2	15 (2.2)
3	9 (1.3)
Non-applicable	298 (44.7)
Permanent needs^a^	
Third-party assistance (partial or total)	71 (10.6)
Regular medical treatments	47 (7.1)
Regular medical appointment	42 (6.3)
Technical aids	38 (5.7)
Drugs	29 (4.4)
Orthoses	16 (2.4)
Consumables	12 (1.8)
Prothesis	11 (1.7)
Others	26 (3.9)
Non-existent	540 (81.0)

^a^Permanent needs are not mutually exclusive.

^b^Percentages may not add up to 100 due to rounding.

In 19 cases, *Future Damage* (FD) was attributed to the increased damage that was expected to occur because of intraarticular fractures (wrist [*n =* 2], hip [*n =* 7], knee [*n =* 8] and ankle [*n =* 3]) and joint instability (shoulder [*n =* 3] and ankle [*n =* 1]). In these cases, it was considered that posttraumatic arthrosis was very likely to develop, and the placement of a total prosthesis would be necessary in the future. In some of these cases, victims had more than one type of FD. In these situations, the PFD was higher (*P =* 0.03) because FD points were added to the PFD.

Among those eligible (*n =* 369), 6.5% (*n =* 24) of victims were considered unable to perform either their usual work (grade 2) or any kind of work (grade 3) (Tables 5 and 8) (*Permanent Professional Repercussions,* PPR). Overall, 19 became unemployed and four retired because of a disability resulting from the RTA. This means that 6.2% (23/369) of individuals became professionally inactive as a result of the RTA. Correlations were found between PPR and all types of severity degrees (all *P <* 0.001), ISS (*P <* 0.001), PFD (*P <* 0.001), and FD (*P =* 0.009). No correlation was observed between PPR and pathological history (*P =* 0.12) or sex (*P =* 0.22).

Table 4 shows the results for *Permanent Aesthetic Damage* (PAD), *Permanent Repercussion on Sexual Activity* (PRSA) and *Permanent Repercussion on Sporting and Leisure Activities* (PRSLA). The distribution of levels of PAD (*n =* 299; 44.8%) was: (a) 1 (*n =* 118; 39.5%), (b) 2 (*n =* 109; 36.5%), (c) 3 (*n =* 39; 13.0%), (d) 4 (*n =* 27; 9.0%), (e) 5 (*n =* 5; 1.7%), and (f) 6 (*n =* 1; 0.3%). For PRSA levels (*n =* 24; 3.6%), the distribution was: (a) 1 (*n =* 2; 8.3%), (b) 2 (*n =* 3; 12.5%), (c) 3 (*n =* 4; 16.7%), (d) 4 (*n =* 7; 29.2%), (e) 5 (*n =* 4; 16.7%), (f) 6 (*n =* 2; 8.3%), and (g) 7 (*n =* 2; 8.3%). For PRSLA (*n =* 66; 9.9%), the distribution was: (a) 1 (*n =* 14; 21.2%), (b) 2 (*n =* 17; 25.8%), (c) 3 (*n =* 10; 15.2%), (d) 4 (*n =* 5; 7.6%), (e) 5 (*n =* 11; 16.7%), (f) 6 (*n =* 8; 12.1%), and (g) 7 (*n =* 1; 1.5%). No significant differences were found between these damage parameters and sex.

Overall, 19% (127/667) of victims were estimated to have permanent needs resulting from the accident (Table 8), and 10.6% (71/667) became dependent on third-party assistance, including 64 (9.6%) being partially dependent and seven (1%) wholly dependent. Most of the victims being dependent on third-party assistance were older people (48/71, 67.6%), and only one (1.4%) was a child, who had a PFD of 100 points because of very severe brain injuries. There was a difference between older people and working-age adults (*P <* 0.001) but no significant difference by sex.

## Discussion and conclusions

### Victims

No differences were found in the sex distribution, which is consistent with some studies [12, 13]. However, others found some differences, albeit some finding male predominance in RTAs [3, 4, 8, 14–22] and other female predominance [23, 24]. The group most commonly affected was working-age adults (65%), followed by older people (27%) [13, 14], which may be related to the different risk exposures of each group. Men were more likely to be younger than women (*P =* 0.01), with more women in the older people group (*P =* 0.05). This is consistent with other work [14, 25], and is probably the result of longer female active life and longevity.

### Accidents

Most RTAs were associated with a vehicle impact (63.4%), most often cars (65.5%), followed by motorcycles (25.8%). Overall, 63.1% of victims were drivers. However, previous studies have found the type of vehicle varies by country and primary mode of transport [3, 4]. Many studies have reported more cars involved in RTAs [4, 17, 19, 21, 25, 26], but in the Netherlands, for example, accidents are more likely to involve bicycles [3, 14], an aspect that can contribute to different RTA outcomes.

The group most involved in collisions was working-age adults, and most of the victims wore protective devices at the time of the accident (87.5%), usually seatbelts (83%) or helmets (100%), as in other studies [27]. For car drivers, there was some variation (82.3%–93%), depending on the existence of a reminder within the vehicle [28].

Accidents involving someone being run over were more frequent in both children and older people than working-age adults (*P =* 0.03 and *P <* 0.001). Age (under 15 and over 75) and physical condition are the primary risk factors for pedestrians [29]. Pedestrians hospitalised after RTAs are often older than both the average road user and motor vehicle occupants who are also hospitalised [30].

### Injuries

Most injuries occurred in the limbs, either lower (53.5%) or upper (49.5%), followed by the head and neck (38.7%), with significant differences among the three age groups, as reported in other studies [8, 14, 18, 19, 22, 23]. This seems to be primarily related to the physical characteristics of the victims, such as size [15], previous health state, and type of accident (collisions or being run over) [4, 24].

Around one-fifth of injuries (21.3%) were severe or critical (ISS ≥ 16), and older people had a significantly higher ISS (*P =* 0.004), consistent with previous studies [14, 15, 25]. This may be because of the poorer physical condition of this age group, making these individuals more vulnerable in trauma cases [31], and to the greater number of accidents involving being run over in this age group. We did not observe any difference between sexes, but some authors have found that women, especially older women, tend to have more serious injuries than men [15, 23]. However, others have found that men have more severe lesions [22]. Studies have also shown that men and women experience accidents differently. Women have a higher risk of permanent disability due to injuries from whiplash (recovering more poorly than men), and body size (which means that the effectiveness of vehicle safety equipment is lower in women, who tend to sit with the seat further forward, making them more susceptible to trauma in the chest and lower limbs). By contrast, men tend to experience more serious injuries, with higher ISS, probably because they suffer more violent/high energy accidents, and are more likely not to wear seat belts [15, 23].

### Temporary outcomes

Temporary outcomes are defined as those that are identified from the moment of the accident until the date when either the injury is deemed to have healed without having resulted in any sequelae or of injury “consolidation” (the date after which no further significant clinical development is expected in terms of sequelae, corresponding to the end of the period of temporary damage) [9]. This period includes any resulting injuries, complications, treatments, hospital stays and sick leave (considering the day-to-day activities and work or training activities), and the physical and psychological suffering inherent in the experience of the trauma and the subsequent process [9].

We found that the average *Temporary Functional Deficit* (length of time during which daily life was impaired) was 199.6 days among working-age adults. This can be considered a relevant period of inactivity or reduced activity. Among older people, this period was longer for *Total Temporary Functional Deficit* (hospitalisation) because of the greater ISS, but lower for *Partial Temporary Functional Deficit.* This is probably because in the recovery period rehabilitation is not as rapid as in working-age adults, because of older people’s lower capacity and need for future physical activities. This means that more older people became dependent on a third-party (26.7%, 48/180), compared with 5.1% (22/431) of working-age adults. Children showed a longer period of *Partial Temporary Functional Deficit*, which may be because in many injuries, especially traumatic brain injuries and lower limb fractures, the medico-legal evaluation is only completed after the end of the pubertal period, which greatly increases the number of days assigned to this damage parameter. This issue needs further study because it reflects an important personal, familial, and economic impact, and it may constitute a specific aspect of medico-legal PIA. Almost no previous studies have examined this issue.

The average length of *Temporary Professional Repercussion* was 171.7 days, which is lower than the results of Murgatroyd (231 days) [21] but still represents an important number of days before a return to work. ISS was significantly linked to the period of recovery time (Table 5) [18, 26]. There were no differences by type of accident and recovery time, despite accidents involving people being run over being more severe (Table 5).

Most victims were assigned QD grades 3 and 4 (78.3%), and only 3.2% had grades 6–7. The only victim with QD 7 was a child with severe neurological, orthopaedic and gastroenterological sequelae, with higher body, functional and situational outcomes and a PFD of 100 points, who was totally dependent on third-party assistance. There were 21 victims with QD 6, mostly related to severe accidents, injuries, and sequelae, and just one who had witnessed the death of a family member during the RTA. Differences were found by QD, ISS, and type of accident, which is a particularly important aspect. This allows us to suggest that, in this study, QD considered aspects related to both accident experience and injury severity, which is consistent with the original concept (Table 1) [9].

### Permanent outcomes

To assure a comprehensive evaluation of permanent outcomes, the 3D methodology for PIA is used in Portugal. In our sample, this evaluation showed the existence of permanent body, functional and situational consequences in 74.7%, 72.6% and 72.0% of cases (Table 6). This showed a good correlation between these outcome levels and both DFP (*P <* 0.001, *P <* 0.001, *P <* 0.001) and PPR (*P <* 0.001, *P <* 0.001, *P <* 0.001). This is fundamental, and allows us to suggest that the damage parameters attributed concretely assess the outcomes reported by the victims, considering their health condition and their daily life and situation.

The most frequent body sequelae were musculoskeletal (64.8%, Table 5) and particularly affected the limbs [32]. This was expected because 73% of accident-related disabilities are attributed to orthopaedic impairment [33]. The functional outcomes (Table 5) were mostly related to posture, dislocations, and transfers (52.0%), which is in line with the most frequent type of body sequelae. It is also consistent with a previous study that found 49% of RTA victims experienced functional activity limitations [33].

Situational outcomes are the consequences for the victims’ daily life. Findings included the following.

1. *Acts of everyday life* (Table 6): 51.7% of the victims reported that their daily life was affected, but the rate and severity of the effect may depend on the ISS and the age group. This issue deserves separate and more detailed analysis. One previous study [22] found that 55% of RTA victims with serious injuries reported an impact on their everyday life, but only 22% of victims with mild-to-moderate injury reported the same impact. These acts of everyday life are one of the aspects considered in PFD evaluation. However, in this study, PFD was attributed to more victims (72.9%), perhaps because its evaluation also considers aspects related to affective and social life and sporting and leisure activities. PFD was correlated with ISS (*P* < 0.001), as expected [18, 22]. Older people had higher PFD values than adults (*P* < 0.001), which is in line with both the ISS, their previous state (pathological and physiological) and the literature [4, 33, 34].
2. *Affective and social life and sporting and leisure activities* (Table 6): 40.5% of victims reported some damage to these aspects of life. Another study [4] indicated that 25.2% of victims reported an impact on affective or family life and 46.9% on leisure or sports activities. These aspects are considered in the PFD evaluation, and may also be included in PAD, PRSA and PRSLA, which were considered present in 44.8%, 3.6%, and 9.9% of the cases in this study. The first two damage parameters showed differences between the age groups (Table 4), as expected, considering the personal lower valorisation of aesthetic and sexual aspects by older persons. The low rate of PRSA (3.6%) is common in the PIA context, primarily because many victims do not disclose this damage, often because they are ashamed to do so [35].
3. *Professional life* (Table 6): 36.4% of victims reported some difficulties, similar to a previous study [4]. The damage parameter that corresponds to this aspect is PPR, which, in this study, was considered present in 34.4% (127/369) of the applicable cases (Table 8). The minimal difference found between these two kinds of evaluations of professional life activities (2%) may be because some victims described complaints that did not have a medical explanation. Overall, 6.5% of victims were considered unable to perform their usual work because of RTA sequelae, compared with a rate of 5%–16% in the literature, varying by the severity of the accident [4, 17, 26]. The literature also shows that success in returning to work after an RTA depends on (a) injury type and severity [4, 16, 17, 21, 26]; (b) occupational skill levels, where low levels are considered a significant risk of a longer time before return to work [17, 21, 26]; and (c) age and sex because older and female victims are more likely to need more time off work following an RTA, with a significant number of older people failing to return to work [26, 36]. Correlations were found between PPR and ISS (*P* < 0.001), PFD (*P* <0.001), and FD (*P* = 0.009) but not between PPR and pathological and traumatological history (*P* = 0.2 and *P* = 0.4).

Damage coefficient, calculated through the 3D methodology, showed that victims (a) recovered fully or with minimal difficulties, but with autonomy and without dependency in 85.3% of cases; (b) were dependent on medication and/or technical aids in 4.0% of cases; (c) were partially dependent on third-party assistance in 9.6% of cases; and (d) totally dependent on a third-party in 1.0% of cases. These results are similar to another study [19], which found that 79.2% of victims experienced full recovery, with 13.2% showing mild disability, 2.9% moderate disability, and 1.1% severe disability. One study stated that 90% of the burden of serious road injuries is due to lifelong consequences that are encountered in 20% of victims [32].

Children exhibited a lower damage coefficient than adults, and older people a higher coefficient (*P <* 0.001; *P <* 0.001). This is consistent with the literature, which suggests that children progress with less disability than adults, but older people experience a greater impact on their health than younger people [4, 20, 23, 37]. Another study [13] reported that very young and old individuals tend to have the highest risk for low quality of life compared to victims between those ages. However, several authors have found that women experience more sequelae than men in all age groups [4, 12, 13, 20, 23], and one study stated that the risk of disability related to traffic injury was higher in women [37].

### Medico-legal personal injury assessment methodology

The Portuguese medico-legal methodology for PIA used in this study considers victims in their real-life situation, which allows a comprehensive assessment, supporting effective and useful damage repair. This is because it uses a 3D description of the permanent outcomes and links that to quantification of temporary and permanent damages, considering several parameters of damage [6–9]. However, despite the relevance of the outcomes that we have described, and the importance of medico-legal assessment and compensation for these damages, there is very little medico-legal literature on this issue. The literature also shows differences in medico-legal PIA methodologies by country [5, 38, 39], which hampers the comparison of results from different studies.

This is true even within Europe, especially between northern and southern countries, because of differences in civil law. However, it is not solely a function of civil law because there are differences even between countries with similar legislation. In Spain, for example, there is a specific law for RTAs, with a medical scale (Act 35/2015, 22 September). This sets out the norms for PIA, including temporary and permanent incapacities, aesthetic damage, dependences, and technical aids. It respects the basic principles of injury compensation, but there are no official assessment guidelines [40–42]. In France, there are several medical scales for permanent incapacity evaluation in civil law [43, 44]. The “*Barème du Concours Médical*” (*Décret* 2003-314, 4^th^ April) [45] is mandatory for PIA ordered by insurance companies. The *Société de Médecine Légale et de Criminologie de France,* in association with the *Association des Médecins Experts en Dommage Corporel,* has also published the “*Barème d’évaluation médico-légale*” , which is more comprehensive and includes scales for assessment of suffering, as well as for aesthetic and sexual damages [46]. However, there are no official assessment guidelines. In Portugal, there are strict and thorough standards, including scales and official tables, for a personal and comprehensive medico-legal evaluation of victims of RTAs, considering both temporary and permanent damage, and both economic and noneconomic aspects [6, 7].

In the USA, the gold standard methodology used for personal damage assessment is the *American Medical Association’s Guides to the Evaluation of Permanent Impairment* [47]. Its aim is to calculate and estimate the percentage of injury suffered by a person caused by trauma or illness that manifests itself as a structural or functional loss in some organic system [48]. In Brazil, the law that established mandatory insurance for RTAs (*Danos Pessoais causados por Veículos Automotores de via Terrestre*, DPVAT) published a table to assess permanent incapacity (Law No. 6194/1974). However, there is no official and systematic protocol for PIA in civil law [49, 50].

These are just some examples demonstrating that each country has its own methodology and tools to assess PIA. Considering these differences and the absence of global criteria for medico-legal PIA, the harmonisation of this practice seems to be an important goal. It would be particularly useful for examiners and would make it possible to perform comparative studies between different populations and samples. This is currently a difficult, if not impossible, task.

These harmonisation attempts have been performed by the *Confédération Européenne d’Experts en Évaluation et Réparation du Dommage Corporel* (CEREDOC) since 1998. Standards and a medical table for the assessment of noneconomic damage were introduced in 2003 and have been updated several times [51]. However, these standards are largely consensual in most countries of Europe and South America [39], and differences persist (e.g. in the terminology used to refer to various parameters of damage, the criteria for assessment and the tables for disability and other damage assessment).

### Results summary

This study revealed that RTA severity was generally serious (ISS mean 9.5), and higher in children and older people. The most frequent body sequelae were musculoskeletal (64.8%), which were associated with functional and situational outcomes (51.7% for acts of everyday life, 40.5% for affective and social life, and sporting and leisure activities, and 36.4% for professional life). Temporary damage resulted in an average length of impairment of daily life of 199.6 days, and required 171.7 days before return to work. The average degree of QD was 3.7/7. Permanent parameters of damage were, on average, 7.3/100 points for PFD, 0.43/3 for PPR, 2/7 for PAD, 3.9/7 for PRSA, and 3.2/7 for PRSLA. Overall, 19% of victims had permanent needs (10.6% needed third-party assistance). These outcomes have significant repercussions for the victim’s life. The Portuguese medico-legal methodology, by considering victims in the context of their everyday life and situation, allows for a comprehensive assessment and supports effective and useful damage repair. The differences among the three age groups and the impact of the more severe cases justify further detailed medico-legal studies in these specific situations on children, older people, and severely injured victims.

### Limitations of this study and further studies

The greatest limitation of this study is that there are no studies with similar methodologies for results comparison. The number of cases among children and older individuals was also significantly lower than among working-age adults, and the sample of severely injured people was also small, which prevented further analysis.

Considering the significant differences found between the three age groups, showing that children and older people have important specificities, we consider that these age groups deserve additional studies. They may even merit the creation of medico-legal guidelines that, to our knowledge, do not currently exist. This may also be true of more severe cases, which deserve deeper and more detailed medico-legal studies.

## Authors’ contributions

Flávia Cunha-Diniz developed the literature review, made the study design and statistical analysis, and wrote the manuscript; Tiago Taveira-Gomes orientated the statistical analysis and supported writing the manuscript; José Manuel Teixeira assisted with the data collection and the discussion of the results; Teresa Magalhães conceived the study, made the medico-legal examinations and the database, and coordinated all the study. All authors contributed to the final text and approved it.

## Data Availability

Results from the dataset are presented in the paper. The full dataset is available from the first author upon request.
